# Supplementary material for: Association of angiotensin-converting enzyme gene insertion/deletion polymorphisms with risk of hypertension among the Ethiopian population
Source: PLoS One. 2022 Nov 10;17(11):e0276021. doi: 10.1371/journal.pone.0276021 (PMC9648817; doi:10.1371/journal.pone.0276021)
Supplement: S1 File — (DOCX) [file pone.0276021.s002.docx]

## Annex 1: Primer specification

## Annex 2: Sample collection and Laboratory procedure

1. **Blood sample collection procedure**
   - - 1. Explain the procedure clearly to participant giving time to ask any questions, ensuring the

patient is comfortable about the procedure.

- - - 1. Obtain informed consent from the participant prior to blood taking.
      2. Ensure all equipment is ready to hand in a tray next to the participant.
      3. Identify a good-sized vein, usually in the antecubital fossae or on the dorsum (back) of the hand.
      4. Apply a tourniquet proximal to the site of venepuncture to ensure engorgement of vein with blood.
      5. Prepare a 10ml syringe with either a green or blue needle depending upon the size of the vein or prepare the Vacutainer with a butterfly needle.
      6. Clean the site of venepunture with an alcohol swab.
      7. Insert needle into vein looking for blood flashback in the bevel of the syringe.
      8. Insert the needle directly into the vein and withdraw peripheral blood of approximately 5 ml transfer the blood in to the purple EDTA tubes slowly. Carefully label the tubes with patient HTN study number and date and time of blood sample was taken. Mark the tubes with HTN study, in order they are readily recognised as this will help with processing at the chief site.
      9. Once enough blood has been withdrawn, undo the tourniquet with the needle still in place.
      10. Take cotton swab and place over site of needle insertion (Venepuncture) and gently remove the needle.
      11. Apply direct pressure with the cotton swab over the puncture site to stem any bleeding. This should be carried out for 2mins, after which the swab should be removed to ensure bleeding has stopped. If not affix the swab with gauze tape.

1. **Laboratory procedure for genetic analysis**

Five milliliter fasting venous blood sample was collected from each participant using EDTA coated tube by following aseptic blood collection procedure. Then, the blood samples were kept in a -4^o^C refrigerator for genetic analysis through the salting out method.

**DNA isolation from peripheral blood salting out method.**

**The genomic DNA Isolation procedure**

**Preparation of Reagents**

- TKM 1 Buffer / Low salt buffer (500 ml):
- 0.605 g of TrisHCl (10mM) pH 7.6,
- 0.372 g of KCl (10 mM),
- 1.016 g of MgCl 2(10 mM),
- 0.372g of EDTA (2mM) was dissolved in 500ml of distilled water
- Triton-X (10ml): Added 0.1 ml of 100 % Triton-X to 9.9ml of distilled water.
- TKM 2 Buffer / High salt buffer(100 ml):
- 0.121 g of TrisHCl (10mM) pH 7.6,
- 0.074 g of KCl (10 mM),
- 1.203 g of MgCl 2(10 mM),
- 0.074 g EDTA (2mM),
- 0.467 g of NaCl (0.4 M) were dissolved in 100ml of distilled water
- 10% SDS: 10gram of sodium dodecyl sulphate was dissolved in 100ml distilled water.
- 6M NaCl: 8.765 g of NaCl was dissolved in 25 ml of distilled water.
- TE Buffer:
- 0.030 g of TrisHCl (10mM) pH 8.0, and
- 0.009 g of EDTA (1mM) were dissolved in 100ml of distilled water
- Isopropanol
- Ethanol (70%)

**Procedure**

**RBC Lysis**

1. Take 300 µl of EDTA blood in an autoclaved 1.5 ml eppendorf tube
2. Add 900 µl of TKM 1 and 50 µl of 1x Triton-X.
3. Incubated at 37^0^ C for 5 minutes to lyses the RBCs.
4. **C**entrifuge at 8000 rpm for 3 minute and discard the supernatant.
5. Repeat this step 2-3 times with decreasing amount of 1x Triton-X till, Until complete lyses of RBC and a white pellet of WBCs remaining.

**Cell Lysis**

1. Add 300 µl of TKM 2 and 40 µl of 10% SDS to the cell pellet, Mix it thoroughly and incubate at 37^0^ C for 5 minutes.
2. At the end of incubation, add 100 µl of 6M NaCl and vortex it to precipitate the proteins.
3. Centrifuge at 8000 rpm for 5 minutes.

**Precipitation of DNA**

1. Transfer the supernatant in to a new eppendorf tube
2. Add 300 µl of isopropanol and inverting the eppendorf slowly to facilitate precipitation.
3. Centrifuge the eppendorfs at 8000 rpm for 5 minutes to pellet down the DNA.
4. Discard the supernatant; add 500 µl of 70% ethanol and mix slowly to remove any excess salts.
5. Centrifuge at 8000 rpm for 5 minutes to pellet down the DNA.
6. Discard the supernatant and air dry the DNA.
7. After thorough drying, 50 µl of TE buffer was added to dissolve the
   DNA and store it -20 ^o^c for the next work.
8. Check the quality and purity of your isolated DNA.
9. The Optical Densities of the DNA samples were obtained by adding 50 µL of the DNA prepared solution into tubes containing 950 µL of diluents.
10. Each of the new solutions is add to quartz cuvette which was placed in a spectrophotometer and the Optical Densities were tabulated using distilled water as the blank control.
11. The 260 nm / 280 nm ratio was obtained to give an analysis of the purity of the sample and the concentration of the extracted DNA present.

**Procedure**

1. Combine the following for each reaction in a 0.2 ml tube:

The PCR reaction mixture (25µl) consisting of 17µl distilled water, 4µl Hot start PCR master mix, 1µl of 10pM of each primer, and 2µl 10ng genomic DNA was prepared. However this reaction may be modified during the optimization of PCR conditions.

| **Components** | **PCR Master mix** | |
| --- | --- | --- |
|  | **For single reaction (μl)** | **10x/ for ten sample (μl)** |
| Hot start PCR master mix | 4 | **40** |
| 10pM of forward primer | 1 | **10** |
| 10pM of reverse primer | 1 | **10** |
| 10ng genomic DNA | 2 | **20** |
| Distilled water | 17 | **170** |
| **Total** | **25µl** | **250µl** |
| **N.B:** For each add 2µl of 10ng genomic DNA to 23µl PCR Master mix +primers +water | | |

1. Prepare a control reaction with no template DNA and an additional 10 μl of sterile water.
2. Run the following program.

**PCR Cycle Settings**

Initial denaturation of the reaction mixture at ----------------95°C for 2 min

Denaturation-------------------------------------------------------94°C for 30 sec

Annealing --------------------------------------------------------- 58°C for 30 sec

Extension---------------------------------------------------------- 72°C for 45 sec

Final extension-----------------------------------------------------72 °C for 9 min

Number of cycle -------------------------------------------------- 30 cycle

Holding temperature -------------------------------------------- -4^o^C

**Agarose Gel Electrophoresis**

**Procedure:**

1. Make a 1- 2% Agarose Gel for electrophoresis.
2. Place 1- 2 drops of ethidium bromide
3. Allow the gel to be solidified and remove the comb.
4. Load 9μL of 100bp DNA ladder in the first well.
5. Mix 3μL of 6x DNA Loading Dye with 12μL of DNA sample and load 15μL into well.
6. Add agarose gel in running container and submerse the in 1x TAE buffer.
7. Run gel at 100V for ~1 hour or until the yellow band of the ladder is near the bottom of the gel (or blue dye band is 1/4 of the way from the bottom)
8. Place gel which is stained with ethidium bromide (0.5 µg/ml) for 30 minutes in a UV box, then photographed on U.V light with digital camera to see the PCR product band.

## Annex 3: Questionnaire (English version)

Dear respondents, you are kindly requested to give correct information accordingly. Thank you for your time and participation.

**I. Personal sociodemographic, anthropometric and clinical information**

Card no._________________

1. Age (in years) _____________

2. Sex: Male Female

3. Educational status: Illiterate

Up to Secondary school

University degree

4. Marital status: Single

Married

Divorced

Widowed

5. Regular physical activity: Yes No

6. Residential area: Urban Rural

7. Height (m) _________________

8. Weight (in Kg) _______________

9. Body Mass Index (kg/ m2) ____________________

10. Waist circumference (cm) ___________

11. Hip circumference (cm) ________________

12. Waist-to-hip ratio_________________

13. Alcohol consumption: Yes No

14. Smoking: Yes No

15. Physical Exercise: Yes No

16. Salt intake (gm/day):___________

17. Family history of hypertension: Yes No

19. Blood Pressure (mmHg), SBP:___________ DBP:_________

20. ACE gene Ploymorphism

II ID DD
