# Supplementary material for: Association of angiotensin-converting enzyme gene insertion/deletion polymorphisms with risk of hypertension among the Ethiopian population
Source: PLoS One. 2022 Nov 10;17(11):e0276021. doi: 10.1371/journal.pone.0276021 (PMC9648817; doi:10.1371/journal.pone.0276021)
Supplement: S1 Raw images — (DOCX) [file pone.0276021.s003.docx]

**Uncropped gel images**

**Image 1:** A sample of original gel/blot image of isolated DNA samples


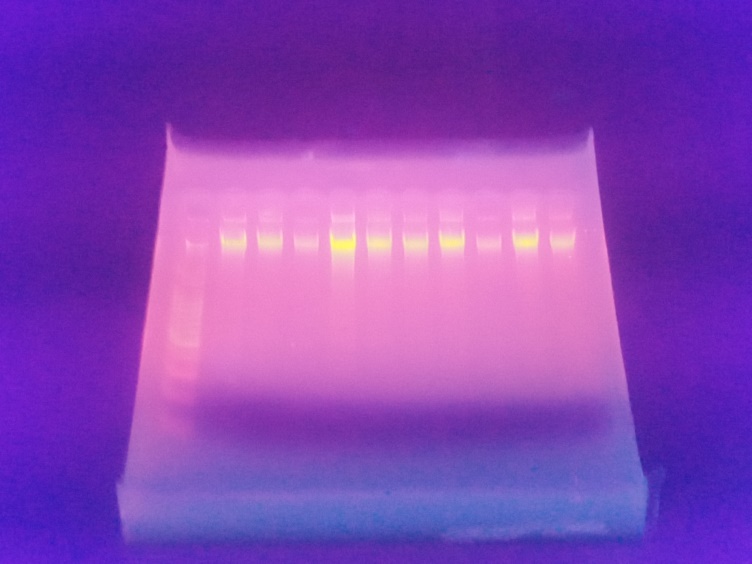


**Image 2:** A sample of Images of amplified DNA samples
