# Supplementary material for: Association of angiotensin-converting enzyme gene insertion/deletion polymorphisms with risk of hypertension among the Ethiopian population
Source: PLoS One. 2022 Nov 10;17(11):e0276021. doi: 10.1371/journal.pone.0276021 (PMC9648817; doi:10.1371/journal.pone.0276021)
Supplement: Attachment — Submitted filename: Response to Reviewers.docx [file pone.0276021.s005.docx]

**Title:** **Association of angiotensin-converting enzyme gene insertion/deletion polymorphisms with risk of hypertension among the Ethiopian population**

**Manuscript ID: PONE-D-22-09370**

**Point by point response to editor and reviewers comment**

Dear Editor/reviewers, we are very grateful for your effective comments given to our manuscript. We have considered your eminent comments and suggestions and modified the manuscript accordingly. The responses are ***highlighted in the main manuscript***.

***Authors’ response:*** ***Dear reviewer, thank you very much for your essential comments for the improvement of the manuscript.***

***The comments are addressed and revised point by point as follows:***

**Review Comments to the Author**

Please use the space provided to explain your answers to the questions above. You may also include additional comments for the author, including concerns about dual publication, research ethics, or publication ethics. (Please upload your review as an attachment if it exceeds 20,000 characters)

**Reviewer #1:** This manuscript presents interesting results, however there are some major flaws with the study. It needs to be proof read and some sections need to be written more concisely. Introduction and discussion portion need to be written strongly. Statistical analysis also has some flaws. Kindly check the attached file for more detailed comments.

**Response for Reviewer #1:** Thank you very much. We have done the proof reading accordingly to have well written manuscript. Some literatures are added in the introduction and discussion section to make the paper stronger. The statistical analysis section have been modified accordingly (**Page 10&11, Table 1 and Page 11&12, Table 2**).

**Reviewer #2:** The manuscript “PONE-D-22-09370” is aimed to find the association of ACE gene insertion/deletion polymorphisms with risk of hypertension among the Ethiopian population. Manuscript is interesting and discussed properly. Some minor changes could attract more audience and more justified.

1. Angiotensin-converting enzyme (ACE) gene insertion/deletion (I/D) polymorphism were reported as a risk factor for other populations as discussed in introduction part of paper, a systemic meta-analysis results would make manuscript interesting.

2. Comparison of genotype of ACE gene insertion/deletion (I/D) with ACE serum level would make results more exciting.

**Response for Reviewer #2:**

1. Yes of course, the ACE gene I/D polymorphism as a risk factor of HTN is studied in different population. But still no study in the current population specifically. And the Meta analysis will be done after the publication of this finding.
2. Really, the comparison of genotype of ACE gene insertion/deletion (I/D) with ACE serum level would give better result and finding. However, unluckily we have no access to the reagents for enzymatic assay and it is already mentioned in the limitation section of the manuscript.

**Reviewer #3:** The manuscript NO. PONE-D-22-09370 is well written and very important for the field, therefore, I recommend the manuscript for publishing but after improving the grammatical and typing error in the manuscript.

**Response to Reviewer #3:** Thank you. We have tried to improve the grammatical and typing error in the manuscript accordingly.

**Reviewer #4:** This is an association study angiotensin-converting enzyme gene insertion/deletion polymorphisms with risk of hypertension among the Ethiopian population. The study included 64 hypertensive patients and 64 controls. While the data is interesting, the manuscript has significant omissions. My primary concerns are the following:

a) The finding is interesting and involves an important public health issue but the key problem with this study is that in the complex phenotype field, few researchers would have confidence in an association that is based on these small sample sizes. (Even if the authors stated as the power of the study).
b) The discussion could be made much more interesting. As it is, they primarily reiterate their results and provide a bit more literature review. However, it would be interesting if they could discuss clinical implications and areas for further study.
c) Authors have listed ACE I/D polymorphism and HTNI reports in different population. I would appreciate if author will provide a table with frequency and association results from different population. It will help readers to have a glance at global variation for the polymorphism association HTN1

**Response to Reviewer #4:** Thank you very much for your constructive comments, we fully agreed with the comments and have been justified the comments below.

a) Even though the sample size was calculated statistically, with this small sample size generalization would be difficult. We have also included it as a limitation and suggested as recommendation better to be done with larger sample size. But still since there is no study conducted in the study area, this finding would be utilized as a baseline data to conduct further advanced studies with lager sample size. The main reason to have small sample size in this study is due to the financial issues and has been recommended for further researchers in the main body of the manuscript (**Page 18, line 381-383**).
b) We have included some clinical implications and areas for further study in the discussion section in order to improve the quality of the paper (**Page 16, line 333-337**).
c) We have attempted to include a table elaborating the distribution of ACE I/D polymorphism in different population and compared it with the present finding (**Page 14, line 291-296 and Page 15, Table 4**).
