# Supplementary material for: Association of angiotensin-converting enzyme gene insertion/deletion polymorphisms with risk of hypertension among the Ethiopian population
Source: PLoS One. 2022 Nov 10;17(11):e0276021. doi: 10.1371/journal.pone.0276021 (PMC9648817; doi:10.1371/journal.pone.0276021)
Supplement: Attachment — Submitted filename: Response to Reviewers.docx [file pone.0276021.s006.docx]

**Title:** **Association of angiotensin-converting enzyme gene insertion/deletion polymorphisms with risk of hypertension among the Ethiopian population**

**Manuscript ID: PONE-D-22-09370R1**

**Point by point response to editor and reviewers comment**

Dear Editor/reviewers, we are very grateful for your effective comments given to our manuscript. We have considered your eminent comments and suggestions and modified the manuscript accordingly. The responses are ***highlighted in the main manuscript***. The comments are addressed and revised point by point as follows.

**Review comments to the Author**

**Reviewer #1:** Authors have included corrections and justified almost all the raised concerns. However, there are few more concerns such as:

1. The primer sequence given to amplify the ACE gene polymorphism gives a PCR product of 191bp and the insertion sequence is 287bp, which makes it 478bp (in case of insertion). Then why 490bp is mentioned in the text?
2. Page 13, line 277: Figure 2 should not be mentioned as it is only representative figure showing different genotypes, not the frequency distribution.
3. Table 3 legend: "Distribution of ACE genotypes and alleles frequency of ACE gene polymorphism (I/D) between HTN and controls at University of Gondar Comprehensive Specialized Hospital, Northwest Ethiopia, 2020/21 (n=128)". There is no need to mention the university name and location in table heading.
4. In Table 3, what does a, b, and c correspond to in various values as well as in p-values?
5. Three Un-cropped gel images for isolated DNA samples have been provided by the authors in supplementary file. However all the three images are same, with different angles and color contrast. And the other two images provided for amplified PCR products, one image is the replicate of representative gel picture in the paper given earlier and the other image is a very bad one for doing genotyping. If these images are the best representative ones then looking at these images raises the question about the technical precision and methods used for PCR as well as gel electrophoresis.

**Response for Reviewer #1:** Thank you very much for your constructive comments. We fully agree with the comments and have justified the comments below.

1. The insertion fragment of PCR product seems to be about 487bp through the mathematical addition of the 190bp and the deleted alu of 287bp. However, previously established method for PCR detection of the insertion/deletion polymorphism of the human ACE gene has determined the fragment and found it to be 490bp (Rigat et al., 1992). It is also supported by several studies (Dai et al., 2019, Aggarwal et al., 2017, Pavlyushchik et al., 2016, Krishnan et al., 2016, Alsafar et al., 2015, Zawilla et al., 2014, Rashed et al., 2015). Moreover, in the present study the band is formed at about 490bp of the reference ladder. That is why we included it in the manuscript's text.
2. Thank you; we agree fully. We have removed it from the text and only Table 3 is mentioned to represent the frequency distribution showing different genotypes. (**Page 13, Line 277**).
3. On the legend of the Table 3, we have included the hospital name "University of Gondar Comprehensive Specialized Hospital" to have an informative title. We have removed it if it is deemed irrelevant. (**Page 14, Line 288**).
4. In Table 3, the superscript a, b, and c labeled on the chi-squared values were used to show the corresponding p-values leveled with corresponding letters (a, b, and c). We reasoned that mentioning the p-values of odds ratio and chi-square in the same table would be confusing to the readers. Therefore, it’s better to use letter labels to allocate the p-values of each chi-squared value. (**Page 14, Line 289**).
5. Yes, of course, there was duplication. Therefore, we have included a single un-cropped gel image for isolated DNA samples and a single gel image of amplified PCR products. Regarding the quality of the gel, of course, these images were not the best representatives of the gel runs we have recorded. However, we didn’t store the images captured during experimental runs and simply included them as it is what is at our hand. (**S1_raw_images**) Moreover, the images included in the manuscript are of better quality, which can resolve the quality concerns.

**References:**

1. AGGARWAL, N., KARE, P. K., VARSHNEY, P., KALRA, O. P., MADHU, S. V., BANERJEE, B. D., YADAV, A., RAIZADA, A. & TRIPATHI, A. K. 2017. Role of angiotensin converting enzyme and angiotensinogen gene polymorphisms in angiotensin converting enzyme inhibitor-mediated antiproteinuric action in type 2 diabetic nephropathy patients. *World journal of diabetes,* 8**,** 112-119.
2. ALSAFAR, H., HASSOUN, A., ALMAZROUEI, S., KAMAL, W., ALMAINI, M., ODAMA, U. & RAIS, N. 2015. Association of Angiotensin Converting Enzyme Insertion-Deletion Polymorphism with Hypertension in Emiratis with Type 2 Diabetes Mellitus and Its Interaction with Obesity Status. *Disease Markers,* 2015**,** 7.
3. DAI, S., DING, M., LIANG, N., LI, Z., LI, D., GUAN, L. & LIU, H. 2019. Associations of ACE I/D polymorphism with the levels of ACE, kallikrein, angiotensin II and interleukin-6 in STEMI patients. *Scientific Reports,* 9**,** 19719.
4. KRISHNAN, R., SEKAR, D., KARUNANITHY, S. & SUBRAMANIUM, S. 2016. Association of angiotensin converting enzyme gene insertion/deletion polymorphism with essential hypertension in south Indian population. *Genes & Diseases,* 3**,** 159-163.
5. PAVLYUSHCHIK, O. O., AFONIN, V. Y., SAROKINA, V. N., CHAK, T. A., KHAPALIUK, A. V. & ANISOVICH, M. V. 2016. Association of the ACE I/D gene polymorphism with DNA damage in hypertensive men. *Cytology and Genetics,* 50**,** 304-311.
6. RASHED, L., ABDEL HAY, R., MAHMOUD, R., HASAN, N., ZAHRA, A. & FAYEZ, S. 2015. Association of Angiotensin-Converting Enzyme (ACE) Gene Polymorphism with Inflammation and Cellular Cytotoxicity in Vitiligo Patients. *PLOS ONE,* 10**,** e0132915.
7. RIGAT, B., HUBERT, C., CORVOL, P. & SOUBRIER, F. 1992. PCR detection of the insertion/deletion polymorphism of the human angiotensin converting enzyme gene (DCP1) (dipeptidyl carboxypeptidase 1). *Nucleic Acids Res,* 20**,** 1433.
8. ZAWILLA, N., SHAKER, D., ABDELAAL, A. & AREF, W. 2014. Angiotensin-converting enzyme gene polymorphisms and hypertension in occupational noise exposure in Egypt. *International journal of occupational and environmental health,* 20**,** 194-206.
