# Supplementary material for: Association of angiotensin-converting enzyme gene insertion/deletion polymorphisms with risk of hypertension among the Ethiopian population
Source: PLoS One. 2022 Nov 10;17(11):e0276021. doi: 10.1371/journal.pone.0276021 (PMC9648817; doi:10.1371/journal.pone.0276021)
Supplement: Attachment — Submitted filename: Response to Reviewers.docx [file pone.0276021.s007.docx]

**Title:** **Association of angiotensin-converting enzyme gene insertion/deletion polymorphisms with risk of hypertension among the Ethiopian population**

**Manuscript ID: PONE-D-22-09370R2**

**A point-by-point response to the editor and reviewer's comment**

Dear Editor/reviewers, we are very grateful for your effective comments given to our manuscript. We have considered your eminent comments and suggestions and modified the manuscript accordingly. The responses are ***highlighted in the main manuscript***. The comments are addressed and revised point by point as follows.

**Response to the Editor:**

Since all the data related to genotype (II, ID, or DD) for all the samples are included in the supplementary file, simply we have taken sample/representative gel images to show how the ACE gene I/D polymorphism of all samples was identified. Moreover, previously published articles, including the one published in this journal (Rashed et al., 2015), have taken representative gel images only and simply included the genotype information of all samples in the tables of supplementary documents. We did the same thing and don't believe it is mandatory to include the gel pictures for all 128 samples because the genotypes of all 128 samples are contained in the supplementary file.

**Review comments to the Author**

**Reviewer #1:** The authors have answered the queries. However, I believe the authors should provide all the raw gel images related to PCR genotyping (for all the samples). It would help to resolve the question of data authenticity.

**Response for Reviewer #1:** Thank you very much for your constructive comments. We fully agree with the comments and have justified the comments below.

1. All the data are fully available within the supplementary document including the ACE gene I/D polymorphism of all 128 samples. However, we have taken representative photographs/images of gel images. Having this, since we have considered the representative gel images/photographs enough, we didn’t capture the images of 128 samples. Simply we have recorded and entered the gene polymorphism data after observing the formation of bands as it is whether at 190bp, 490bp, or on both 190bp and490bp. Simply we have taken a sample/representative gel image to show how the ACE gene I/D polymorphism of all samples was identified. But the types of ACE gene I/D polymorphism of all samples could be obtained in the supplementary document attached to the journal. Therefore, the presence of all the raw gel images for all the samples may not be mandatory and could not significantly affect the quality of the paper.

Furthermore, previously published articles, including those published in PLOS ONE (Rashed et al., 2015), simply have included a sample of gel image of PCR amplified products. They didn’t include the gel images of all the samples they performed and they have included the genotypes in the supplementary document.

**Reference:**

RASHED, L., ABDEL HAY, R., MAHMOUD, R., HASAN, N., ZAHRA, A. & FAYEZ, S. 2015. Association of Angiotensin-Converting Enzyme (ACE) Gene Polymorphism with Inflammation and Cellular Cytotoxicity in Vitiligo Patients. *PLOS ONE,* 10**,** e0132915.
